# Host attraction and host feeding patterns indicate generalist feeding of *Culex pipiens* s.s. and *Cx. torrentium*

**DOI:** 10.1186/s13071-024-06439-7

**Published:** 2024-08-30

**Authors:** Magdalena Laura Wehmeyer, Linda Jaworski, Hanna Jöst, Tatiana Șuleșco, Leif Rauhöft, Sara M. Martins Afonso, Markus Neumann, Konstantin Kliemke, Unchana Lange, Ellen Kiel, Jonas Schmidt-Chanasit, Felix Gregor Sauer, Renke Lühken

**Affiliations:** 1 Bernhard Nocht Institute for Tropical Medicine, Hamburg, Germany; 2 Carl Von Ossietzky University, Oldenburg, Germany; 3 Ministry of Social Affairs, Health and Sports Mecklenburg-Vorpommern, Werderstraße 124, 19055 Schwerin, Germany; 4 Faculty of Mathematics, Informatics and Natural Sciences, Universität Hamburg, 22609 Hamburg, Germany

**Keywords:** Mosquito, Host attraction, Host feeding patterns, *Culex pipiens* biotype *pipiens*, *Culex pipiens* biotype *molestus*, *Culex pipiens* hybrid biotype *pipiens* × *molestus*, *Culex torrentium*

## Abstract

**Background:**

Mosquito host feeding patterns are an important factor of the species-specific vector capacity determining pathogen transmission routes. *Culex pipiens* s.s./*Cx. torrentium* are competent vectors of several arboviruses, such as West Nile virus and Usutu virus. However, studies on host feeding patterns rarely differentiate the morphologically indistinguishable females.

**Methods:**

We analyzed the host feeding attraction of *Cx. pipiens* and *Cx. torrentium* in host-choice studies for bird, mouse, and a human lure. In addition, we summarized published and unpublished data on host feeding patterns of field-collected specimens from Germany, Iran, and Moldova from 2012 to 2022, genetically identified as *Cx. pipiens* biotype *pipiens*, *Cx. pipiens* biotype *molestus*, *Cx. pipiens* hybrid biotype *pipiens* × *molestus*, and *Cx. torrentium*, and finally put the data in context with similar data found in a systematic literature search.

**Results:**

In the host-choice experiments, we did not find a significant attraction to bird, mouse, and human lure for *Cx. pipiens* *pipiens* and *Cx. torrentium*. Hosts of 992 field-collected specimens were identified for Germany, Iran, and Moldova, with the majority determined as *Cx. pipiens* *pipiens*, increasing the data available from studies known from the literature by two-thirds. All four *Culex pipiens* s.s./*Cx. torrentium* taxa had fed with significant proportions on birds, humans, and nonhuman mammals. Merged with the data from the literature from 23 different studies showing a high prevalence of blood meals from birds, more than 50% of the blood meals of *Cx. pipiens* s.s. were identified as birds, while up to 39% were human and nonhuman mammalian hosts. *Culex torrentium* fed half on birds and half on mammals. However, there were considerable geographical differences in the host feeding patterns.

**Conclusions:**

In the light of these results, the clear characterization of the *Cx. pipiens* s.s./*Cx. torrentium* taxa as ornithophilic/-phagic or mammalophilic/-phagic needs to be reconsidered. Given their broad host ranges, all four *Culex* taxa could potentially serve as enzootic and bridge vectors.

**Graphical Abstract:**

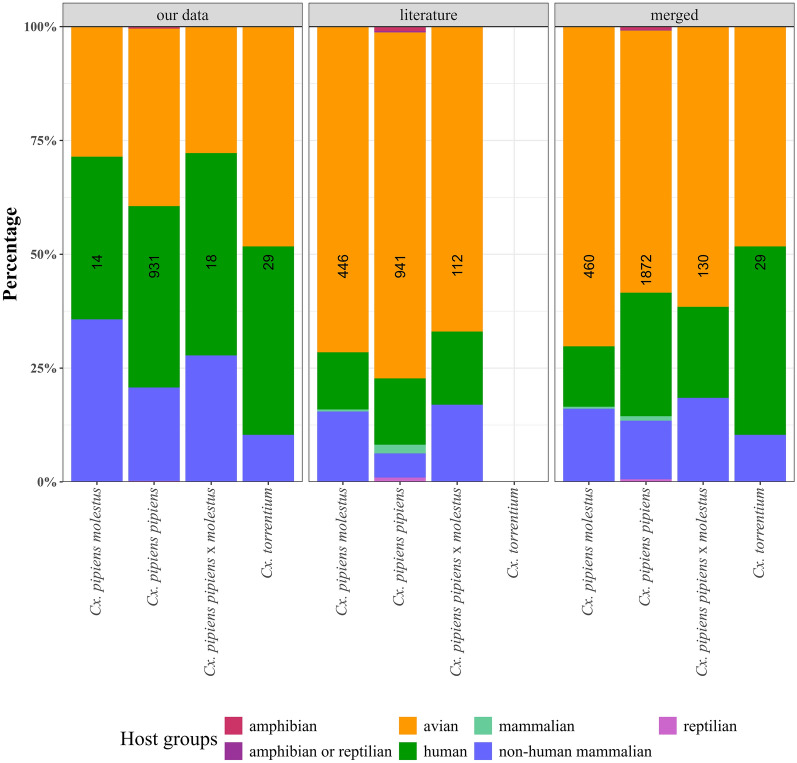

**Supplementary Information:**

The online version contains supplementary material available at 10.1186/s13071-024-06439-7.

## Background

Host feeding patterns describe an important component of vector capacity, i.e., the probability of a vector–host contact [1]. This interaction is essential to understanding pathogen transmission cycles, e.g., to identify potential vector species [2]. Host feeding patterns of mosquitoes are characterized by intrinsic (genetic) and extrinsic (environmental) factors [3–5]. Intrinsic factors are considered the main drivers of host preference for mosquito species with a narrow range of host species, e.g., high preference of *Culex territans* or *Uranotaenia unguiculata* for amphibians [6, 7], while extrinsic factors are expected to be relevant for species with a broad range of host species, e.g., host availability for *Cx. pipiens* [8].

It is proposed that specialists evolve when there is a fitness gain achieved by consuming one optimal host compared with feeding on a range of suboptimal hosts [9]. In contrast, generalists are expected to occur in environments with a low probability of host encounter, and the advantage of waiting for an optimal host is weighed against the risk of death prior to blood feeding and reproduction [1]. To understand the transmission cycle of mosquito-borne pathogens, it is important to accurately describe species-specific differences in host feeding patterns, as it enables the classification of mosquito species as enzootic vectors or bridge vectors of a given pathogen, e.g., *Cx. torrentium* is considered the enzootic vector (bird–mosquito–bird) of Sindbis virus, while *Aedes cinereus* the bridge vector (bird–mosquito–human) [10].

Misconceptions about mosquito host feeding patterns are deeply rooted in the literature. One prominent example is *Cx. pipiens* s.s./*Cx. torrentium*, including the taxa *Cx. pipiens* biotype *pipiens* (*Cx. pipiens pipiens*), *Cx. pipiens* biotype *molestus* (*Cx. pipiens molestus*), the hybrid between both biotypes *Cx. pipiens* biotype *pipiens* × *molestus* (*Cx. pipiens pipiens* × *molestus*), and *Cx. torrentium*. The females cannot be identified by classic morphology [11], but the taxa differ considerably in their ecology [12–15]. In the literature, *Cx. pipiens pipiens* and *Cx. torrentium* are commonly described as ornithophilic/-phagic [13, 16–18], while there is no unified definition for this terminology other than feeding “often” or preferring to feed on the respective host group compared with other host groups without a defined threshold [19]. In contrast, *Cx. pipiens molestus* is predominantly considered mammalophilic/-phagic [20]. The hybrid between both biotypes with an intermediate host feeding pattern is considered to function as bridge vectors for zoonotic diseases in Northern America [21]. In contrast, recent studies from Europe and Asia show opportunistic host feeding patterns for *Cx. pipiens* s.s./*Cx. torrentium* with a considerable proportion of mammals, including humans. There might be no taxa-specific association with one host group and the taxa have to be considered both potential enzootic and bridge vectors [22–25].

*Culex pipiens* s.s./*Cx. torrentium* are potential vectors of different mosquito-borne pathogens with a high relevance for veterinary and public health. This also applies to Germany, Moldova, and Iran, which are examined in more detail in the present study. *Culex pipiens* s.s./*Cx. torrentium* is widespread in each of the three countries [22, 23], and field-collected specimens are regularly found to be positive for arboviruses as well as their vector competence was confirmed in the laboratory, for example, Usutu virus or West Nile virus [26–32]. This is also reflected in the published information on the host feeding patterns for the countries, which showed that *Cx. pipiens* s.s./*Cx. torrentium* have to be considered potential bridge vectors feeding on birds and mammals, including humans [22, 23]. Nevertheless, although there are several other studies analyzing the host feeding patterns of *Cx. pipiens* s.l. with more than 20,000 identified blood meals all over the world, many studies did not differentiate between the members of the species complex [33].

Therefore, the aim of this study was to provide comprehensive insight into the host feeding patterns of *Cx. pipiens pipiens*, *Cx. pipiens molestus*, *Cx. pipiens pipiens* × *molestus*, and *Cx. torrentium* by (1) analyzing the host attraction of *Cx. pipiens pipiens* and *Cx. torrentium* in a host-choice experiment, (2) summarizing the published and unpublished host feeding patterns for specimens collected in field studies over the last decade analyzed with the same laboratory protocols, allowing for a comparability of the results between Germany, Moldova, and Iran, and (3) finally comparing our results on the host feeding patterns of these taxa with those previously described in the globally available literature.

## Methods

### Experiment on the host attraction of *Cx. pipiens pipiens* and* Cx. torrentium*

*Culex pipiens* s.s./*Cx. torrentium* were reared from egg rafts collected in Weinheim, Germany (49.54° N, 8.66° E) between May and August 2020 using gravid-trap bins baited with a yeast hay infusion. About 1–5 egg rafts were placed in larval rearing trays (22 cm × 15 cm × 7 cm) containing 1 L of tap water. Larvae were fed daily with a small amount of crushed flake fish food (TetraMin Flakes, Tetra GmbH, Melle, Germany). Larval rearing was conducted at 22–26 °C and 40–60% relative humidity. Emerging adults were maintained in 32.5 cm × 32.5 cm × 32.5 cm screened cages under the same temperature and relative humidity conditions and were daily provided with 10% sucrose solution ad libitum. Females used in the host selection trials emerged 4 days prior and deprived of sucrose solution 12 h prior to testing.

**Fig. 1:**
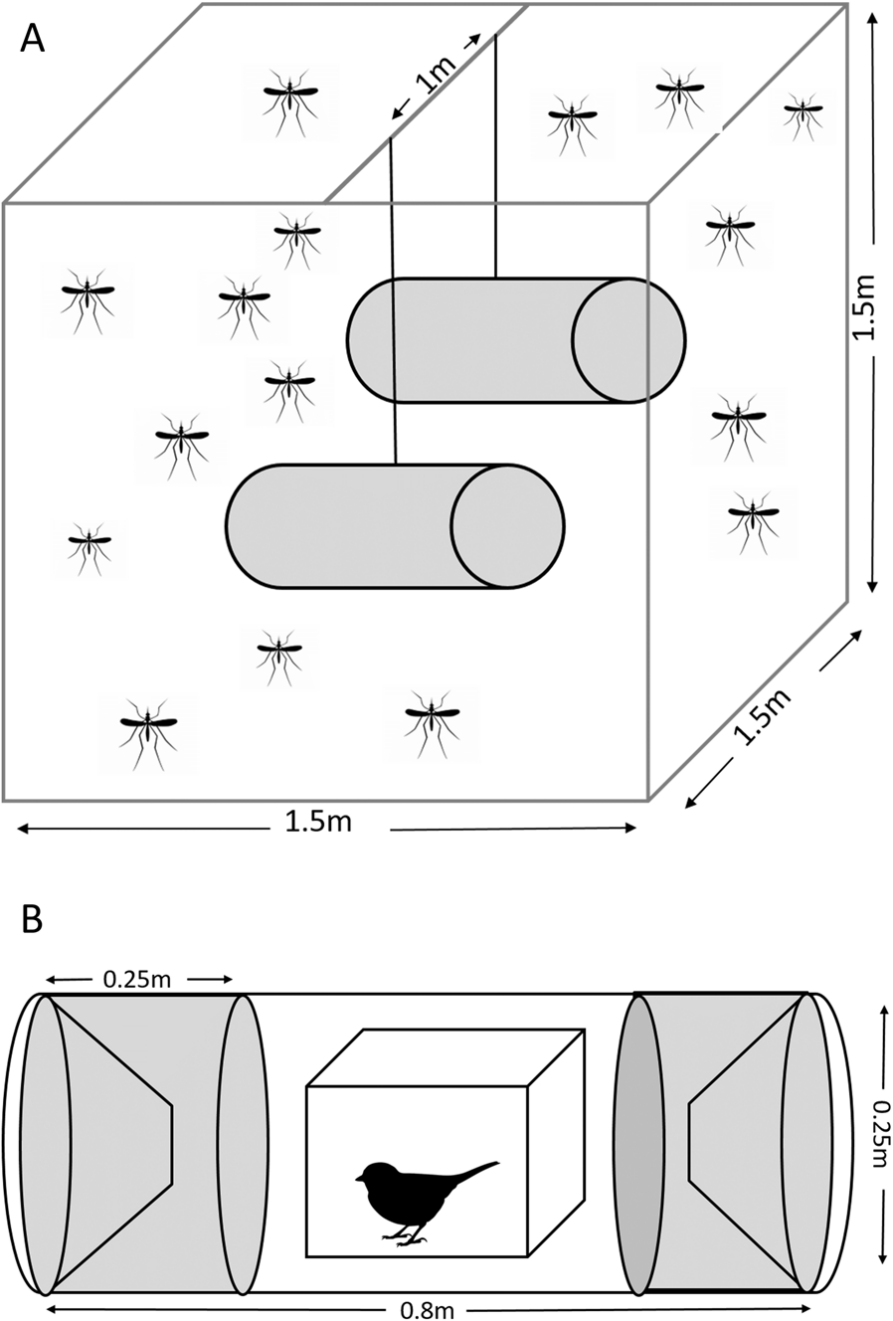
**A** Mesh enclosure with two lard can traps each equipped with an animal or attractant, mosquito pictogram taken from © clipart-library, **B** lard can traps included in the mesh enclosure (Fig. 1); bird pictogram taken from © clipart-library

The trials were conducted with two animals: one grey canary (*Serinus canaria* form *domestica*) and one house mouse (*Mus musculus*). In addition, as an attractant that mimics human skin scents, a packet of BG-Sweetscent (Biogents, Regensburg, Germany) was used with 25 ml CO_2_/min, which is similar to the amount of CO_2_ emitted by the mouse. The CO_2 _emission of the canary (9.22 ml CO_2_/min (SD 1.09) and the mouse (24.82 ml CO_2_/min (SD 1.64) was previously measured with a CO_2_ monitor (AIRCO2NTROL 5000, TFA Dostmann, Wertheim-Reicholzheim, Germany). For this purpose, the individual animals were placed in a box (32 × 25 × 37 cm) and the CO_2_ content was measured before adding the animal and after 10 min. The experiment was repeated three times. A 1.5 m × 1.5 m mesh enclosure was placed inside the laboratory and two lard can traps (25 × 25 × 80 cm) were hung side-by-side separated by one meter [34] (Fig. 1). The lard can traps were constructed from a large tube (⌀ 25 cm) covered at both ends with removable sampling devices with mesh funnels that allowed mosquitoes to enter but prevented them from escaping the tube. A cage with the attractant was placed inside the tube. Trials were performed with the following combinations inserted within the lard can traps: bird–bird, bird–lure, bird–mouse, lure–lure, mouse–lure, and mouse–mouse. The animal or attractant was randomly assigned to one of the lard can traps. Each trial was repeated five times.

*Culex pipiens* s.s./*Cx. torrentium* females entered the trap through one of two removable funnels on either end of the trap. The funnels contained a mosquito-proof mesh that prevented direct contact between the animals and mosquitoes. The trials were conducted from 6 pm to 8 am with an average of 122 females (between 43 and 212 females) for each trial, depending on the availability of 4-day-old females. Mosquitoes in the lard can traps and the remaining mosquitoes in the mesh enclosure were removed with a manual mouth sucking aspirator, stored separately in tubes at −20 °C. All specimens were identified as *Cx. pipiens pipiens*, *Cx. pipiens molestus*, *Cx. pipiens pipiens* × *molestus*, or *Cx. torrentium* using a molecular DNA typing assay [12].

Host attraction was analyzed using individual binomial generalized linear models (GLM) per combination of hosts and mosquito species. The proportions of host-seeking female mosquitoes per lard can trap (from now on “attraction”) was used as response variable (*N* = 10 per GLM) and animal/attractant as two-factorial explanatory variable, e.g., “bird” and “mouse.” Mosquitoes that did not enter one of the lard can traps were not considered as host-seeking and were excluded from the statistical analysis.

### Analysis of the host feeding patterns of *Cx. pipiens* s.s./*Cx. torrentium* collected in Germany, Moldova, and Iran

Our field data on the host feeding patterns of *Cx. pipiens* s.s./*Cx. torrentium* combine previously collected data by us during field studies conducted in Germany [22] and Iran [23] and new, unpublished data collected in different sampling campaigns between 2012 and 2022 in Germany and Moldova. All specimens from the already published studies, as well as the newly collected specimens, were analyzed with the same laboratory workflow [22, 23]. This allows for a better comparability between the results from the three countries, for example, polymerase chain reaction (PCR) primers have been shown to have different specificity [35], potentially influencing the sensitivity for different host taxa between different studies. Sampling sites in all of the three countries covered different dominant land-use categories from urban over rural to natural in each of the countries [22, 23], although an analysis of the differences in host feeding patterns between different land-use categories were not in focus of this study, as it was shown to have no statistically significant impact in our previous studies in Germany [22] and Iran [23]. Mosquitoes were collected with pop-up garden bags as artificial resting sites using a hand-held aspirator [36] or within a nationwide mosquito and pathogen surveillance program using CO_2_-baited Heavy Duty Encephalitis Vector Survey traps (BioQuip Products, Rancho Dominguez, California, USA), Centers for Disease Control miniature light trap (BioQuip Products, Rancho Dominguez, California, USA), and Biogents Sentinel or BG-Pro traps (Biogents, Regensburg, Germany). The collected mosquitoes were left in the trap bags and stored at −20 °C prior to analysis. Each specimen was morphologically identified under permanent cooling [37].

Whole blood-engorged, morphologically identified *Cx. pipiens* s.s./*Cx. torrentium* specimens were placed individually into 2 ml tubes and about 20 pieces of 2.0 mm zirconia beads (BioSpec Products, Bartlesville, USA) as well as 1 ml of cell culture medium (high-glucose Dulbecco’s modified Eagle’s medium; Sigma-Aldrich, St. Louis, MO, USA) were added. The homogenization was performed with a TissueLyser or TissueLyser II (Qiagen, Hilden, Germany) for 2 min at 50 oscillations/s. After clarifying by centrifugation for 1 min at 8000 rpm and 4 °C, the suspension was transferred to a new safe-lock tube. DNA was extracted from 200 μl of the homogenate using the KingFisher™ Flex Magnetic Particle Processor with the MagMAX™ Pathogen ribonucleic acid/DNA Kit (both Thermo Fisher Scientific, Waltham, MA USA).

Two primer sets targeting the cytochrome *b* or *16S* rRNA gens were used [38, 39] following the previously published protocol [22, 23]. All amplicons were further processed with Sanger sequencing (LGC Genomics, Berlin, Germany), sequences pre-processed with Geneious^®^ 7.1.9 [40], and finally compared with GenBank sequences (http://blast.ncbi.nlm.nih.gov/Blast.cgi). Host species were determined using a 95% threshold for percentage identity. Using the same template, all morphologically identified *Cx. pipiens* s.s./*Cx. torrentium* specimens were identified as *Cx. pipiens pipiens*, *Cx. pipiens molestus*, *Cx. pipiens pipiens* × *molestus*, or *Cx. torrentium* using a molecular DNA typing assay [12].

Differences in the proportion for the avian, human, and nonhuman mammalian host feeding groups were evaluated among the three countries by the test of equal or given proportions (*prop.test*) in R (Version: 4.2.2) [41].

### Global literature review on the host feeding patterns of *Cx. pipiens* s.s./*Cx. torrentium*

Data on host feeding patterns were extracted for *Cx. pipiens pipiens*, *Cx. pipiens molestus*, *Cx. pipiens pipiens* × *molestus*, or *Cx. torrentium* from publications identified in a systematic search on 17 June 2024 using the PubMed database with the following strategy: '(Mosquito*[Title] OR Culici*[Title] OR Aedes[Title] OR Culex[Title] OR Anoph*[Title] OR "west nile virus"[Title]) AND (Blood*[Title] OR meal*[Title] OR feed*[Title] OR host*[Title] OR preference*[Title] OR pattern*[Title] OR forage*[Title])'. The methods were described in detail by Wehmeyer et al. [33]. In short, two researchers independently screened the publications for suitability on the basis of following inclusion criteria: (1) the study was conducted in the field, (2) studies using vertebrate baits were included only if mosquitoes had no direct contact with the host or were collected before biting, and (3) ingested blood was analyzed using serological or molecular methods. Studies that were only based on behavior observation, laboratory-reared mosquitoes, or laboratory-based feeding experiments were excluded. For this publication, studies were included where *Cx. pipiens* s.s./*Cx. torrentium* were identified as *Cx. pipiens pipiens*, *Cx. pipiens molestus*, *Cx. pipiens pipiens* × *molestus*, or *Cx. torrentium* using a molecular DNA typing assay. All possible information given on mosquito, detected host taxa, and country were collected and merged into a single database. Blood meal hosts were further categorized into the host groups avian, amphibian or reptilian, reptilian, amphibian, mammalian, human, and nonhuman mammalian.

### Data analysis

All computational analysis was performed in R (Version: 4.2.2) using the R-Studio IDE (Version: 2022.12.0) [41]. Additionally, functions from the following packages were used for data preparation and visualization: dplyr [42], ggplot2 [43], tidyverse [44], readxl [45], stringr [46], plyr [47], and magrittr [48].

## Results

### Experiment on the host attraction of *Cx. pipiens pipiens* and* Cx. torrentium*

**Fig. 2:**
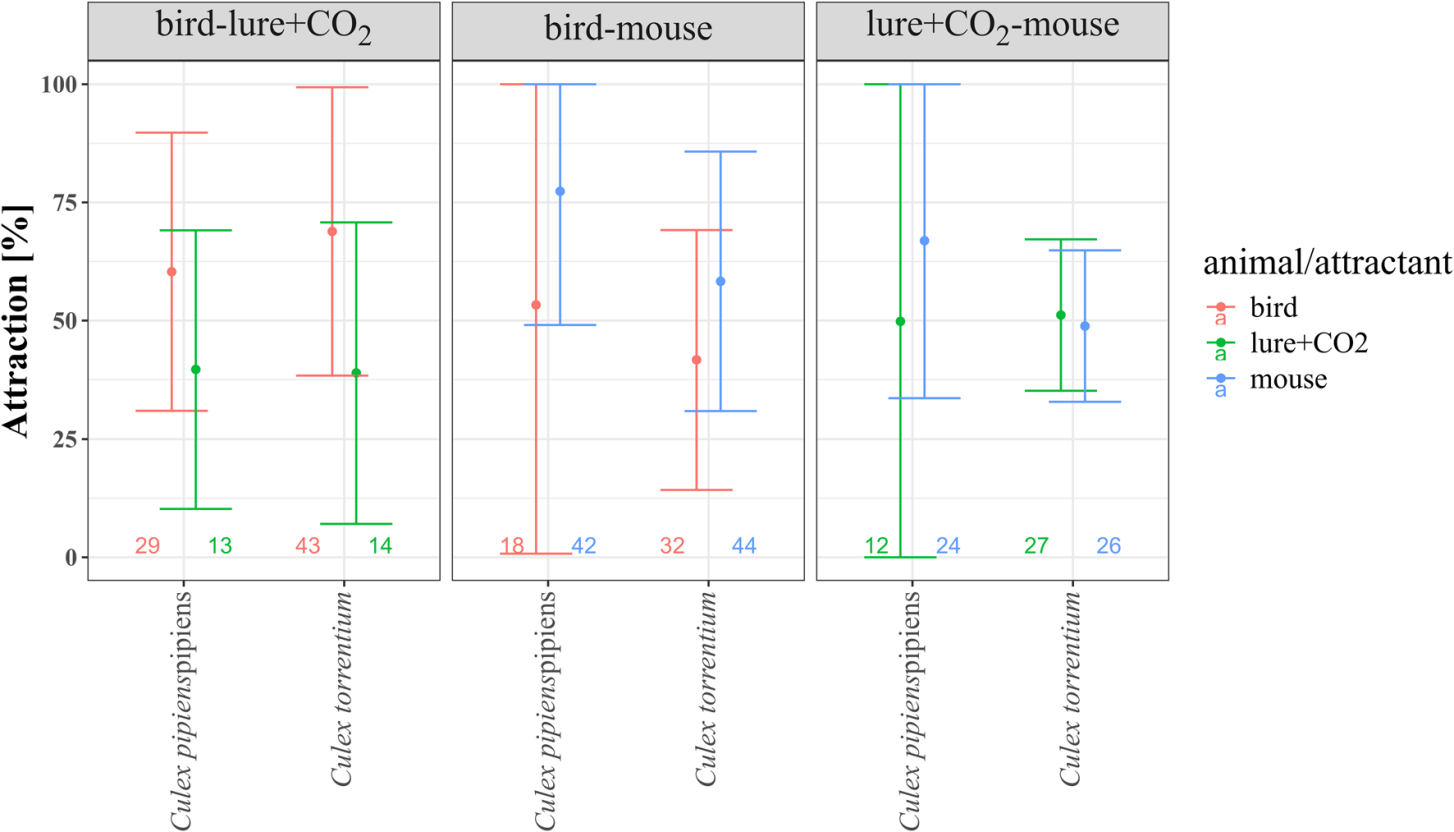
Mean attraction with 95% confidence interval for host/attractant for *Culex pipiens pipiens* and *Culex torrentium*. Numbers on the bottom indicate the total number of specimens collected in the specific lard can trap over five replicates

A total of 268 *Cx. pipiens pipiens* and 350 *Cx. torrentium* females were used in the experimental trials comparing the proportional attraction for bird versus lure, bird versus mouse, and mouse versus lure. Both species showed a higher mean attraction for birds compared with lure with a mean of 60.3% [95% confidence interval (95% CI) 30.9–89.8%] against 39.7% (95% CI 10.2–69.1%) for *Cx. pipiens pipiens* and 58.9% (95% CI 38.4–99.4%) against 38.9% (95% CI 7.1–70.8%) for *Cx. torrentium*. For the trial bird against mouse it was the other way around with a higher mean attraction for mouse against bird with a mean of 53.3% (95% CI 0.7–100.0%) against 77.3% (95% CI 49.1–100.0%) for *Cx. pipiens pipiens* and 41.7% (95% CI 14.2–69.1%) against 58.3% (95% CI 30.9–85.8%) for *Cx. torrentium*. No clear pattern regarding the mean values was observed for the trial lure versus mouse. The 95% confidence intervals of mean attraction for the different trials were highly overlapping (Fig. 2) and neither species showed any statistically significant difference for a host or attractant (binomial GLMs, *P* > 0.05). In addition, no statistical pattern was observed for the same host/attractant in both lard can traps (Additional file 1: Fig. S1).

### Analysis of the host feeding patterns of *Cx. pipiens* s.s./*Cx. torrentium* collected in Germany, Moldova, and Iran

**Fig. 3:**
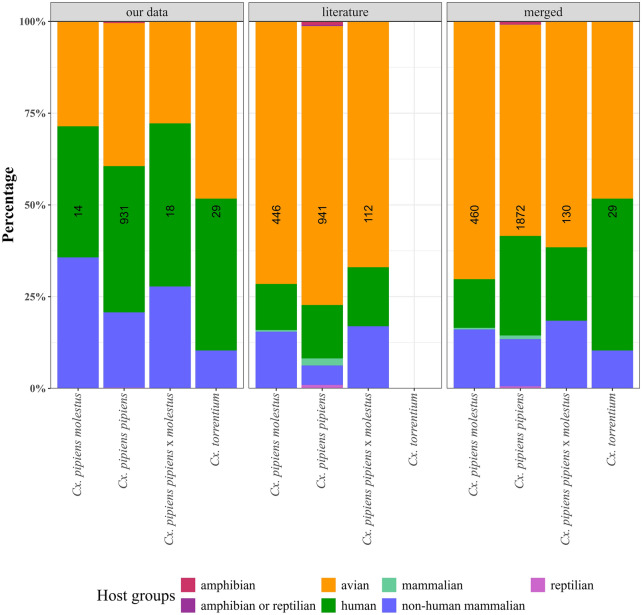
Proportion of host groups detected for *Cx. pipiens molestus*, *Cx. pipiens pipiens*, *Cx. pipiens pipiens* × *molestus*, and *Cx. torrentium*. Data collected in our studies (left), data from literature (middle), and both datasets merged (right). Numbers in the bar indicate the number of blood meals per taxon and dataset. The host group “mammalian” is used if studies do not identify the mammalian species

The host species were identified for a total of 931 blood-fed *Cx. pipiens pipiens*, 29 *Cx. torrentium*, 18 *Cx. pipiens pipiens* × *molestus*, and 14 *Cx. pipiens molestus* collected in Iran, Moldova, and Germany (Fig. 3). For *Cx. pipiens pipiens*, blood meals from human (371, 39.8%) and avian hosts (363, 39.0%) were detected in the highest numbers, followed by non-mammalian hosts detected with 191 blood meals (20.5%) and 4 amphibian blood meals (0.4%). Blood meals of *Cx. torrentium* were dominated by birds (14, 48.3%) and humans (12, 41.4%), while only 3 blood meals (10.3%) were observed from nonhuman mammalian taxa. *Culex pipiens pipiens* × *molestus* fed on humans (8, 44.4%) and showed equal proportions of avian and non-human mammalian blood meals (5, 27.8%). Finally, for *Cx. pipiens molestus*, blood meals from human (5, 35.7%) and non-human mammals (5, 35.7%) were equally frequently detected, shortly followed by avian hosts (4, 28.6%).

**Fig. 4:**
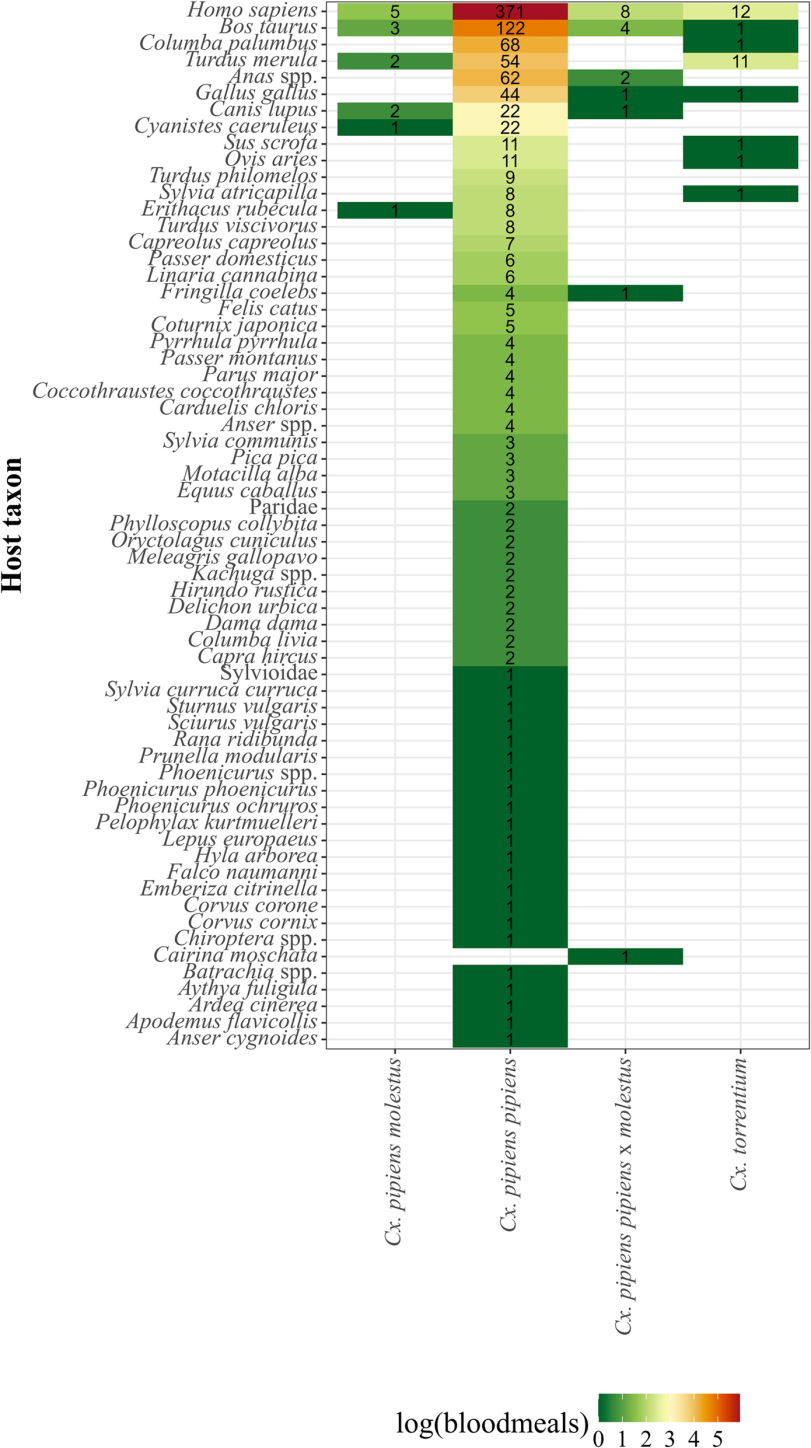
Number of blood meals per host taxon detected in our studies for *Cx. pipiens molestus*, *Cx. pipiens pipiens*, *Cx. pipiens pipiens* × *molestus*, and *Cx. torrentium*

As demonstrated above, a high prevalence of humans is evident for all four studied *Culex* taxa (> 35%, Fig. 4). Focusing exclusively on *Cx. pipiens pipiens* with a sufficient sample size, further frequent host taxa were *Bos taurus* (122 blood meals, 13.1% of all blood meals for this taxon), *Columba palumbus* (68, 7.3%), *Anas* spp. (62, 6.7%), *Turdus merula* (54, 5.8%), and *Gallus gallus* (44, 4.7%). The other blood meals (210, 22.6%) were distributed over many less frequent hosts dominated by different bird species and domestic animals (e.g., *Canis lupus*, *Felis catus*). Comparing the host feeding patterns for the three countries in comparison with the remaining two, a significant lower proportion of nonhuman mammals was observed for Germany (Germany versus Iran: *χ*^2^ = 33.1, *df* = 1, *P* < 0.001; Germany versus Moldova: *χ*^2^ = 6.3, *df* = 1, *P* < 0.012; Iran versus Moldova: *χ*^2^ = 0.27, *df* = 1, *P* = 0.6), while we found lower proportions of humans in Moldova (Germany versus Iran: *χ*^2^ = 2.7, *df* = 1, *P* = 0.09; Germany versus Moldova: *χ*^2^ = 13.2, *df* = 1, *P* < 0.001; Iran versus Moldova: *χ*^2^ = 18.8, *df* = 1, *P* < 0.001) and lower proportions of birds in Iran (Germany versus Iran: *χ*^2^ = 42.1, *df* = 1, *P* < 0.001; Germany versus Moldova: *χ*^2^ = 2.8, *df* = 1, *P* < 0.09; Iran versus Moldova: *χ*^2^ = 29.7, *df* = 1, *P* < 0.001).

For 41 *Cx. pipiens pipiens* specimens (4.4%), two different hosts were detected: 35 mixed blood meals with human and avian blood, 3 with avian and nonhuman mammalian blood, 2 specimens fed on a human and a nonhuman mammal, and 1 specimen contained blood of a bird and an amphibian. One *Cx. torrentium* specimen (3.4%) contained blood from *Homo sapiens* and *Sus scrofa*.

### Global literature review on the host feeding patterns of *Cx. pipiens* s.s./*Cx. torrentium*

We found a total of 23 publications on host feeding patterns that used molecular assays to differentiate *Cx. pipiens pipiens*, *Cx. pipiens molestus*, *Cx. pipiens pipiens* × *molestus*, and *Cx. torrentium* (5 × USA [49–53]; 4 × Japan [54–57] [50–52, 54–56]; 3 × Spain [25, 58, 59]; 2 × each for Australia, Portugal, and UK [17, 60–64]; and 1 × each for Argentina, Iran, the Netherlands, Romania, and Russia [18, 65–68]). When this dataset was merged with our dataset, 1872 identified blood meals were available for *Cx. pipiens pipiens*, 460 for *Cx. pipiens molestus*, and 130 for *Cx. pipiens pipiens* × *molestus* (Fig. 3). No additional data from the literature were available for *Cx. torrentium*. Compared with the new data presented in this study for Germany, Iran, and Moldova with blood meals from birds < 50%, the three *Cx. pipiens* taxa in the merged dataset had more than 50% blood meals from birds, while human and mammalian species each had less than 30%.

**Fig. 5:**
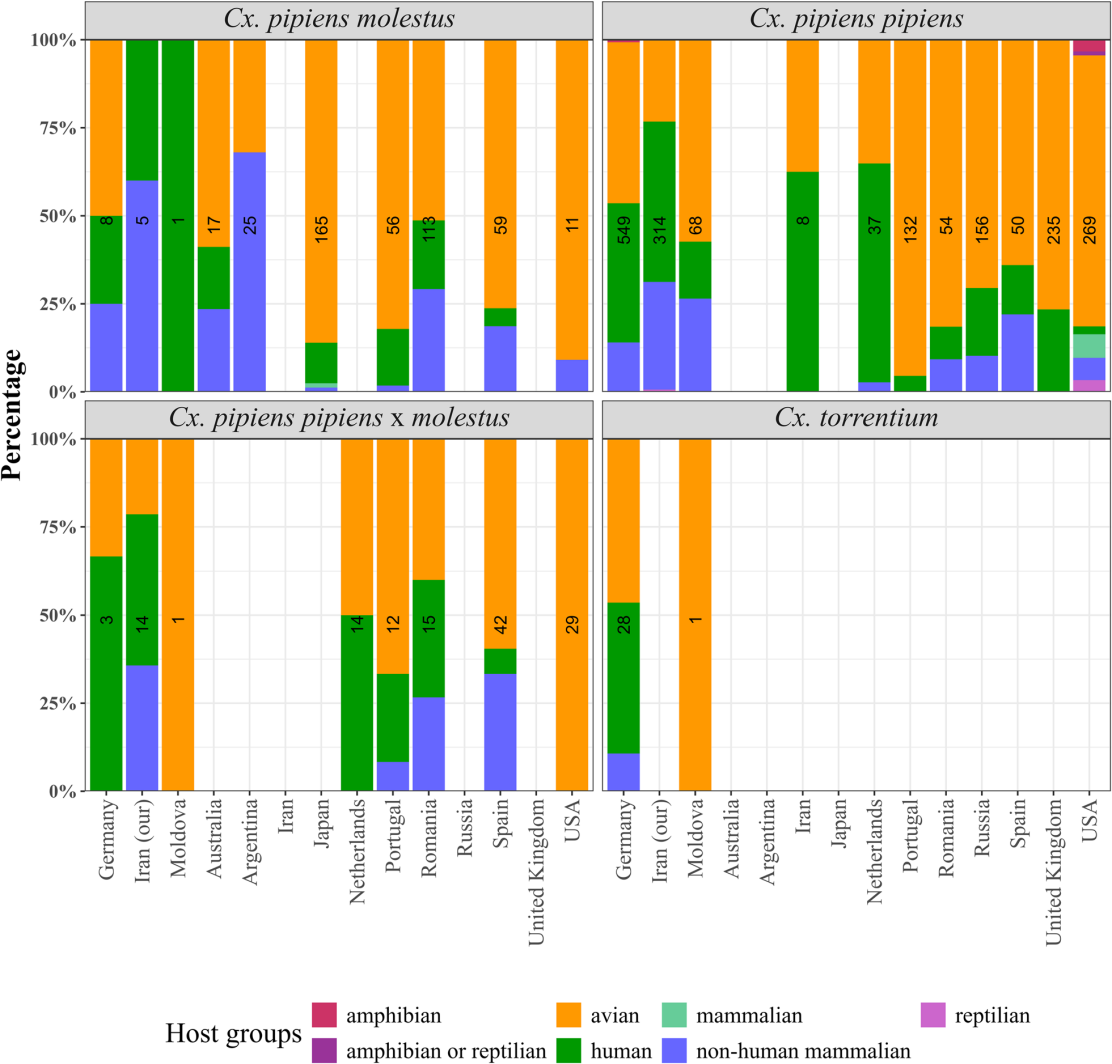
Proportion of host groups for *Cx. pipiens molestus*, *Cx. pipiens pipiens*, *Cx. pipiens pipiens* × *molestus*, and *Cx. torrentium* per country. Data combined blood meals collected by us (Germany, Iran, Moldova) and data from the literature. Numbers in the bar indicate the number of blood meals per taxon and country. The host group “mammalian” is used if studies do not identify the mammalian species

Results from the different countries were heterogeneous. Studies from Romania, the USA, and Portugal showed that *Cx. pipiens pipiens* predominantly fed on birds, with up to 95.5% (Fig. 5). In contrast, higher proportions of mammalian taxa were observed for the newly collected data from Moldova and Germany (42.7% and 35.5%, respectively), and even reached 64.9% and 75.8% in the Netherlands and Iran, respectively. Similarly, low proportions of mammalian hosts were observed for *Cx. pipiens molestus* in the USA, Spain, Japan, and Portugal (< 25%); around half of the feeds in Germany, Australia, and Romania; and a high proportion of 68% in Argentina. The few specimens from Iran and Moldova did not contain any avian blood. For *Cx. pipiens pipiens* × *molestus*, a dominance of mammals was found for Germany, Iran, the Netherlands, and Romania (> 50%); less than 50% for Portugal and Spain; and only blood meals from birds in the USA.

## Discussion

Due to their wide distribution, abundance, and vector competence for WNV, USUV, or SINV, *Culex pipiens pipiens*, *Cx. pipiens molestus*, and *Cx. torrentium* are potentially important vectors of arboviruses in Europe [26–30]. The transmission cycles promoted by these vectors are shaped by their host-feeding patterns, i.e., maintaining enzootic cycles within one host group (e.g., birds) or leading to a spill-over from one host group to another.

We did not observe a significant attraction for mouse, grey canary, or human lure for *Cx. pipiens pipiens* and *Cx. torrentium*. In similar experiments conducted in the USA, *Cx. pipiens pipiens* showed a significant attraction for birds against mammals [69, 70]. For the USA it is especially discussed that hybridization between *Cx. pipiens pipiens* and *Cx. pipiens molestus* is the driver of host attraction with intermediate host acceptance for the hybrid taxon [70]. However, we did not find any differences in the host attraction between *Cx. pipiens pipiens* and *Cx. torrentium* either, which do not hybridize.

Host feeding patterns can differ from host choice experiments under laboratory conditions, that is, they are expected to depend on the availability and abundance of the hosts [8]. Many studies have been conducted worldwide to identify the blood hosts of more than 20,000 *Cx. pipiens* specimens [33], but only a few have differentiated the bioforms of *Cx. pipiens* s.s., and none included *Cx. torrentium*. Nevertheless, in the literature, *Cx. pipiens pipiens* is regularly referred to as ornithophilic/-phagic, whereas *Cx. pipiens molestus* is described as mammalophilic/-phagic or anthropophilic/-phagic [16–18, 71]. Unfortunately this terminology is not based on a standardized classification and is generally used without a clear definition [19].

Studies from the literature differentiating *Cx. pipiens* s.s./*Cx. torrentium* were collated here and showed that *Cx. pipiens pipiens* fed predominantly on avian hosts. Much less data were available for *Cx. pipiens molestus* and *Cx. pipiens pipiens* × *molestus*, but showed a similar pattern with a high proportion of birds. No data were available for *Cx. torrentium*. Nevertheless, there were considerable differences between the countries, with some combinations of countries and taxa reaching more than 62% mammalian hosts, for example, *Cx. pipiens pipiens* collected in the Netherlands [67] and *Cx. pipiens molestus* collected in Argentina. Additionally, for the field-collected specimens analyzed in our laboratory, a broad host use was observed with up to 50% mammalian hosts. The reasons for these differences can be manifold. First, only very few studies differentiated the *Cx. pipiens* s.s./*Cx. torrentium*. Worldwide, more than 20,000 undifferentiated *Cx. pipiens* specimens were analyzed and revealed a broad host feeding pattern with one-third of the blood meals from each human, avian, and nonhuman mammalian host [33]. Our studies on the host feeding patterns in Germany, Iran, and Moldova increased the total number of available taxa-specific information on the host feeding patterns of *Cx. pipiens* s.s./*Cx. torrentium* by two-thirds. Another factor might be the species identification of the different *Cx. pipiens* s.s./*Cx. torrentium* taxa, that is, *Cx. pipiens* s.s. host attraction is considered to be the result of genetic introgressive hybridization between *Cx. pipiens pipiens* and *Cx. pipiens molestus* populations [25]. In addition, host availability is often assumed to drive the host feeding patterns observed in the field [8], but this information is mostly not collected in the field. Our data from Germany, Iran, and Moldova analyzed with the same laboratory workflow showed statistically significant differences for the proportions of the different host groups, e.g., lower proportion of nonhuman mammals for Germany or lower proportion of birds for Iran. However, the underlying drivers of these differences remain unclear and need further evaluation in further work. Our previous studies in Germany and Iran showed that land-use as most obvious driver might not explain these differences in host feeding patterns [22, 23].

The birds mainly detected in blood meals of *Cx. pipiens pipiens* belonged especially to the species *Gallus gallus Columba palumbus, Hirundo rustica,*, and *Turdus merula.* The latter was also present in the feeds of *Cx. pipiens molestus Cx. pipiens pipiens* × *molestus* and dominated the feeds of *Cx. torrentium*. Of these bird species, especially the blackbird *Turdus merula* in particular is known to be part of the transmission cycle of WNV and USUV in Europe, as it was found to die in large numbers during USUV outbreaks [72–74]. At the same time, we observed considerable proportions of human hosts for each *Culex* taxon, highlighting their potential role as enzootic and bridge vectors.

In the field-collected *Culex* specimens analyzed in our laboratory, mixed blood meals were detected in 41 *Cx. pipiens pipiens* and one *Cx. torrentium* specimen. Up to now, only a few mixed blood meals have been described in the literature, for example, for *Cx. pipiens pipiens* or *Cx. pipiens molestus* [17, 49]. The detection of mixed blood meals is interesting information, as it is evidence of the transmission potential transmission risk between two host species. However, the frequency of mixed blood meals must be interpreted with caution. Generally, gel PCRs with subsequent Sanger sequencing were used to identify the blood meal hosts. Different primers have been shown to have different specificity [35], potentially influencing the sensitivity for different host taxa. The presence of gene fragments of two or more hosts could lead to overlapping signals after sequencing, which are difficult to distinguish from low-quality signals, for example, requiring advanced techniques using next-generation sequencing [75]. Thus, actual amounts of specimens with ingested blood of more than one host could be higher than observed.

## Conclusions

*Cx. pipiens pipiens*, *Cx. pipiens molestus*, and *Cx. torrentium* were found to feed with a significant proportion on each avian, human and nonhuman mammalian host. Thus, the classification of *Cx. pipiens pipiens* and *Cx. pipiens molestus* as strictly ornithophilic/-phagic and anthropo- or mammalophilic, respectively, should be reconsidered. The broad host range of these taxa combined with a high vector competence suggests a high relevance as both enzootic and bridge vectors in the transmission cycles of various mosquito-borne pathogens, for example, WNV, USUV, and SINV [26–30]. At the same time, we observed significant differences between data collected from different countries. Future studies especially should focus on the underlying intrinsic and extrinsic factors, e.g., the influence of population genetics, host availability, or general environmental conditions on the host feeding patterns.

### Supplementary Information

Additional file 1: Figure S1. Mean attraction with 95% confidence interval for host/attractant for *Culex pipiens pipiens* and *Culex torrentium*. Numbers on the bottom indicate the total number of specimens collected in the specific lard can trap over five replicates.

## Data Availability

All data are available in the manuscript and in the supplementary files.
